# Leptomeningeal Involvement in Relapsed Multiple Myeloma With Thoracic Spine and Orbital Metastases: A Case Report

**DOI:** 10.7759/cureus.86601

**Published:** 2025-06-23

**Authors:** Dania Abuhalima, Maha Akkawi, Razan Odeh

**Affiliations:** 1 Department of Scientific Research and Medical Projects, An-Najah National University, Nablus, PSE; 2 Department of Medicine, An-Najah National University, Nablus, PSE; 3 Department of Pathology, An-Najah National University Hospital, Nablus, PSE; 4 Department of Hemato-Oncology, An-Najah National University Hospital, Nablus, PSE

**Keywords:** central nervous system involvement, leptomeningeal multiple myeloma, multiple myeloma, orbital metastasis, relapsed myeloma

## Abstract

Multiple myeloma (MM) is a clonal plasma cell disorder that primarily originates in the bone marrow and rarely presents as extramedullary disease. Central nervous system involvement, particularly in the form of leptomeningeal disease, is a rare and aggressive manifestation of MM. We report a case of a 44-year-old female with relapsed MM who presented with headache, diplopia, and ptosis. Brain magnetic resonance imaging revealed multiple enhancing dural nodular lesions. Cerebrospinal fluid cytology was negative for malignant cells. A dural biopsy confirmed leptomeningeal involvement of MM through histopathological examination and CD138 immunostaining. The patient was managed with systemic and intrathecal chemotherapy and corticosteroids and was referred for radiotherapy. Leptomeningeal involvement in MM remains a diagnostic and therapeutic challenge due to its rarity, rapid progression, and nonspecific presentation. This case highlights the need for heightened clinical suspicion and comprehensive diagnostic evaluation in MM patients with neurological symptoms.

## Introduction

Multiple myeloma (MM) is a mature B-cell neoplasm characterized by the presence of ≥10% clonal plasma cells in the bone marrow and evidence of end-organ damage, such as hypercalcemia, renal insufficiency, anemia, one or more osteolytic lesions on skeletal radiography, computed tomography (CT), or positron emission tomography-computed tomography (PET-CT); or myeloma-defining biomarkers (clonal bone marrow plasma cell percentage ≥60%, serum free light chain ratio ≥100, or two or more lesions of at least 5 mm on whole-body magnetic resonance imaging (MRI)) [1].

About 90% of MM patients develop bone lesions, making it the most common malignancy affecting the skeleton following metastasis [2]. Extramedullary involvement of MM is infrequent (about 5% of patients), and central nervous system (CNS) involvement - occurring in approximately 1% of cases - is a rare manifestation that may include malignant plasma cells in the cerebrospinal fluid (CSF) or be diagnosed through imaging and biopsy findings. It can present as localized intraparenchymal lesions, solitary cerebral plasmacytoma, or leptomeningeal multiple myeloma (LMM) [3,4].

Although acquiring LMM in an existing myeloma patient is extremely rare (<1%), its incidence has increased over the last decade - most likely due to increased use of MRI and improved survival of MM patients. However, the prognosis for LMM remains poor, with an overall median survival of two months from the time of diagnosis [5].

## Case presentation

The patient is a 44-year-old female diagnosed with MM in June 2023, after presenting with back pain and being found to have a plasmacytoma involving the T3 vertebra. She received six cycles of VRD (Velcade, Revlimid, dexamethasone) chemotherapy, completing the final cycle in October 2023. Bone marrow studies showed only 5% plasma cells, and spinal MRI demonstrated a significant reduction in the previously identified dorsal lesion one month later.

After remission, she was a candidate for bone marrow transplantation and received high-dose cyclophosphamide as a conditioning regimen prior to the procedure. She recovered smoothly, with continued follow-up by her primary physician.

A follow-up PET scan in May 2024 revealed regression of previously seen mildly hypermetabolic lytic lesions, with residual mild uptake in the left iliac lesion and associated morphologic regression. Maintenance therapy with lenalidomide and zoledronic acid was initiated. Zoledronic acid was used as a bone-modifying agent to manage skeletal involvement.

In June 2024, due to persistent spinal involvement, she underwent partial corpectomy at T3 and T1-T10 spinal fixation. Bone marrow evaluation at that time showed 25% kappa-restricted plasma cells and an elevated IgG level of 2260 mg/dL. At diagnosis, serum protein electrophoresis showed an M spike of 24 g/dL. Serum free light chain analysis revealed an elevated kappa light chain of 432 mg/L and lambda of 16 mg/L, with a kappa/lambda ratio of 27. Immunofixation electrophoresis (IFE) confirmed the presence of IgG kappa monoclonal protein. Cytogenetic studies, including FISH (Fluorescence In Situ Hybridization), were not performed at the time of diagnosis due to limited resource availability.

**Figure 1:**
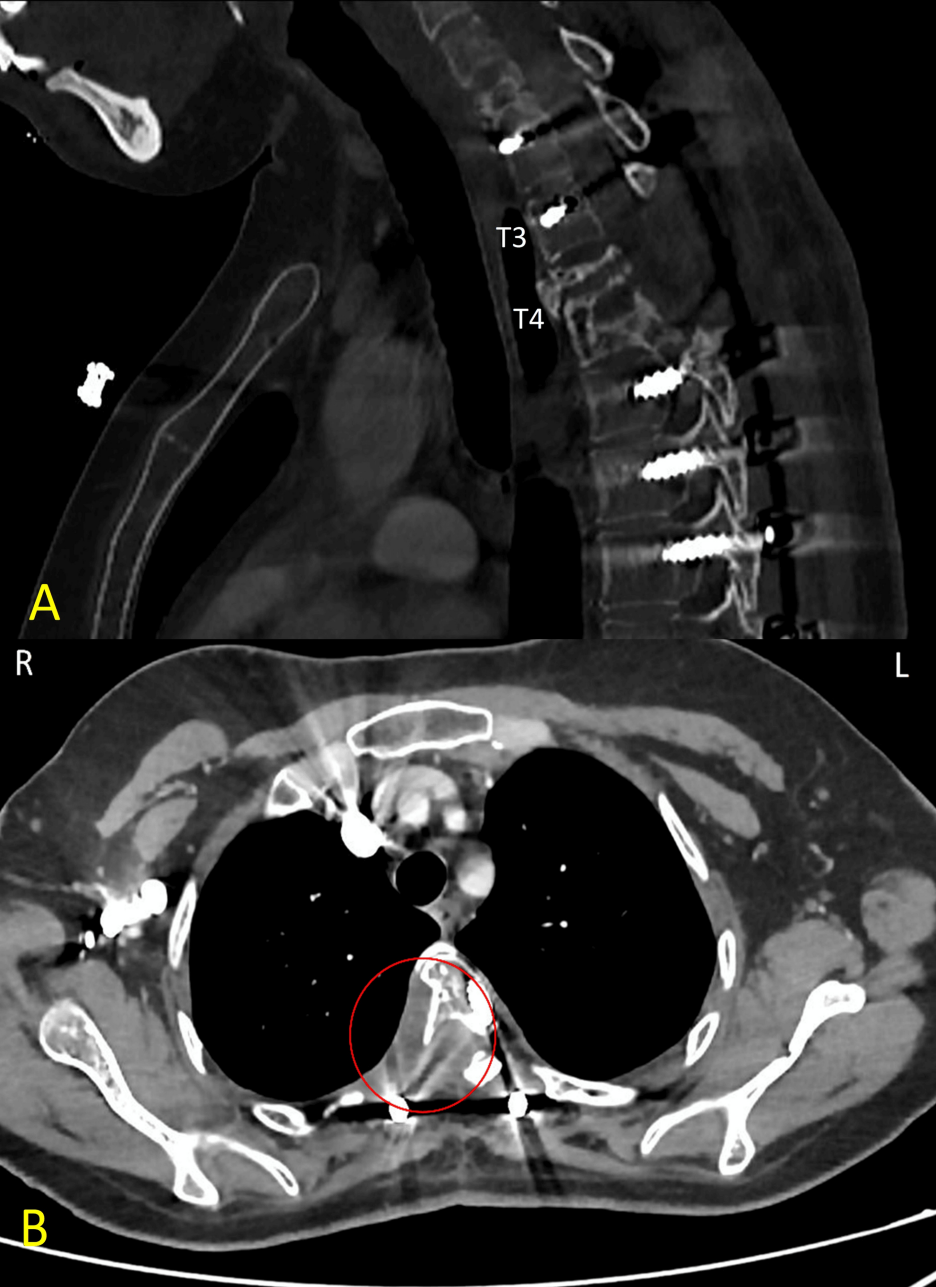
Sagittal and Axial Chest CT Scans (A) Sagittal chest CT showing destructive bone lesions in the T3 and T4 vertebral bodies with associated soft tissue component. (B) Axial chest CT demonstrating possible right-sided pleural invasion (circle) and involvement of adjacent structures. CT, computed tomography

In November 2024, the patient presented with increasing back pain. A CT revealed a destructive, enhancing mass involving the upper thoracic spine - primarily the T3 and T4 vertebral bodies - with significant height loss of the T3 vertebral body. The mass extended to the paraspinal area, predominantly on the right side, involving the T3 and T4 ribs and regional paraspinal muscles, and causing indentation and possible invasion of the right upper lung pleura and lobe. No radiological evidence of spinal cord compression was observed (Figure 1).

Further MM workup at the time confirmed relapsed disease, with significantly elevated IgG levels and bone marrow studies showing 60% plasma cells. The patient was started on VCD (Velcade, cyclophosphamide, and dexamethasone) chemotherapy and referred to our hospital for continued care.

**Figure 2:**
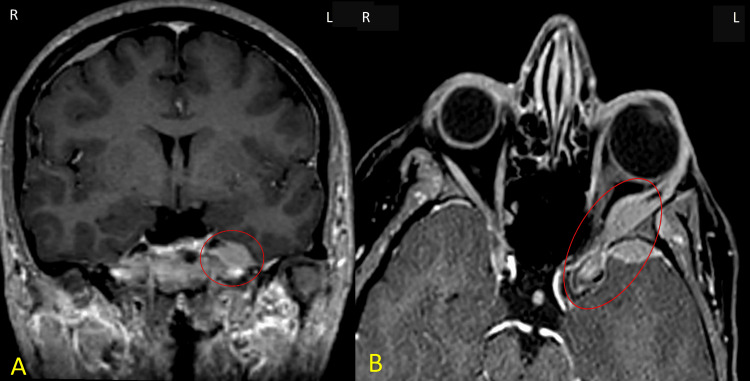
MRI of CNS and Orbital Involvement in Multiple Myeloma (A) Axial post-contrast T1-weighted MRI showing a homogeneously enhancing dural-based lesion (circle) adjacent to the left cavernous sinus and anterior temporal lobe. (B) Coronal post-contrast T1-weighted orbital MRI showing an intraorbital lesion (circle) causing mass effect on the distal aspect of the left optic nerve. MRI, magnetic resonance imaging; CNS, central nervous system

Upon presentation, the patient complained of headache and diplopia with ptosis (sixth nerve palsy). A CT scan of the brain was unremarkable, but an MRI demonstrated multiple scattered, enhancing focal dural nodular thickenings, the largest measuring approximately 0.9 cm adjacent to the left cavernous sinus. Another lesion, measuring 0.5 × 1.3 cm, was observed in the left lateral intra-orbital compartment, exerting mass effect upon the optic nerve. The findings were consistent with dural metastatic deposits. The brain parenchyma and CSF spaces appeared otherwise normal (Figure 2).

**Figure 3:**
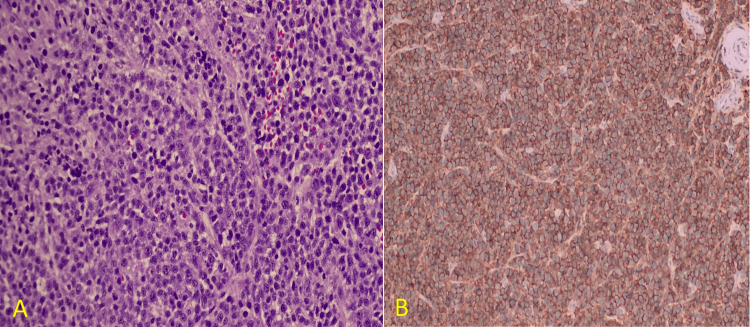
Meningeal Biopsy Confirming Leptomeningeal Myeloma (A) Hematoxylin and eosin (H&E) staining shows sheets of plasma cells with predominantly plasmacytic morphology. (B) CD138 immunostaining highlights the plasma cell sheets, confirming leptomeningeal involvement of multiple myeloma.

CSF analysis showed normal glucose and protein levels, with no malignant cells on cytology; thus, a meningeal biopsy was performed, and histopathological examination revealed sheets of plasma cells with predominantly plasmacytic morphology. Immunohistochemical staining for CD138 confirmed the presence of plasma cell sheets, consistent with MM’s leptomeningeal involvement (Figure 3).

After the diagnosis of LMM, she was admitted for conservative management with dexamethasone and pain management, alongside triple intrathecal chemotherapy, and was referred for local radiotherapy, which she subsequently received.

## Discussion

LMM is a rare and devastating manifestation of CNS involvement in MM, occurring in less than 1% of cases [3]. It typically arises in the context of relapsed or refractory disease, often signifying advanced and aggressive disease biology. Our patient, a 43-year-old female, initially responded well to VRD induction therapy and autologous stem cell transplantation. At relapse, she had markedly elevated IgG (2260 mg/dL) and bone marrow infiltration of 60% plasma cells. Clinically, she presented with headache, diplopia, and ptosis. Although CSF cytology was negative, MRI revealed dural-based lesions, and a meningeal biopsy confirmed LMM.

The differential diagnosis for her cranial nerve palsy and dural lesions included infectious meningitis, meningeal carcinomatosis, and lymphoma. However, the radiologic features and histopathological findings supported a diagnosis of LMM.

While CNS involvement in MM can take various forms, including solitary plasmacytomas, dural-based masses, or intraparenchymal lesions, leptomeningeal spread is the least common and most ominous [5]. The clinical presentation is often non-specific, with headache, visual disturbances, altered mental status, and cranial nerve palsies being the most frequently reported features [5]. Our patient presented with diplopia and headache, and MRI revealed dural-based nodular lesions and an intraorbital mass, which are consistent with meningeal myelomatous involvement.

Diagnosis remains challenging due to the limited sensitivity of CSF cytology, which can be negative in up to 40% of cases [6]. In our case, CSF analysis did not reveal malignant cells, and the diagnosis was confirmed via a meningeal biopsy, which showed CD138-positive plasma cell infiltration indicating LMM. CSF flow cytometry, which may offer higher sensitivity in detecting malignant plasma cells, was not performed, as the initial CSF sample was negative and further analysis was deferred.

The rarity of LMM and the lack of standardized treatment guidelines make management particularly difficult. Therapeutic strategies typically involve systemic chemotherapy, intrathecal therapy, corticosteroids, and radiotherapy [7]. Our patient received triple intrathecal therapy, in addition to steroids and radiotherapy. Despite aggressive intervention, the prognosis remains extremely poor, with a median overall survival of about two to four months following diagnosis [8]. The poor outcomes reflect both the aggressive disease biology and the challenges of delivering effective treatment across the blood-brain barrier.

Although daratumumab-based therapy has shown promise in treating high-risk and relapsed MM [9], it was not initiated in our patient due to financial constraints and limited drug availability in our setting. In the West Bank, Palestine, national treatment guidelines currently do not recommend daratumumab as a first-line agent, further limiting its routine use in clinical practice. This highlights important real-world challenges faced in low-resource environments.

Upfront cytogenetic risk stratification could have further informed treatment intensity, and may have supported consideration of a four-drug induction regimen, including daratumumab - particularly in settings with high-risk features. Unfortunately, such testing was not available at the time of diagnosis.

This case underscores the importance of maintaining a high index of suspicion for CNS involvement in relapsed or refractory MM, especially in the presence of new neurological symptoms. Early MRI evaluation and consideration of biopsy are crucial when CSF findings are inconclusive. While LMM remains a rare complication, its incidence may rise with improved imaging modalities and longer MM survival.

## Conclusions

LMM is an extremely rare and aggressive manifestation of relapsed disease, with a poor prognosis. This case emphasizes the importance of considering CNS involvement in MM patients presenting with neurological symptoms, even with negative CSF cytology. Advanced imaging and histopathological confirmation remain critical for diagnosis. Early recognition and timely intervention through systemic and intrathecal chemotherapy, corticosteroids, and radiotherapy may help improve symptom control, although the prognosis remains poor.
